# Effect of Extending the Original CROSS Criteria on Tumor Response to Neoadjuvant Chemoradiotherapy in Esophageal Cancer Patients: A National Multicenter Cohort Analysis

**DOI:** 10.1245/s10434-020-09372-y

**Published:** 2020-11-28

**Authors:** Helena Hong Wang, Ellen C. de Heer, Jan Binne Hulshoff, Gursah Kats-Ugurlu, Johannes G. M. Burgerhof, Boudewijn van Etten, John Th. M. Plukker, Geke A. P. Hospers, the Dutch Upper Gastrointestinal Cancer Audit (DUCA) Group

**Affiliations:** 1 Department of Surgical Oncology, University of Groningen, University Medical Center Groningen, Groningen, The Netherlands; 2 Department of Medical Oncology, University of Groningen, University Medical Center Groningen, Groningen, The Netherlands; 3 Department of Radiology, University of Groningen, University Medical Center Groningen, Groningen, The Netherlands; 4 Department of Pathology and Medical Biology, University of Groningen, University Medical Center Groningen, Groningen, The Netherlands; 5 Department of Epidemiology, University of Groningen, University Medical Center Groningen, Groningen, The Netherlands

## Abstract

**Background:**

Extending the original criteria of the Chemoradiotherapy for Oesophageal Cancer followed by Surgery Study (CROSS) in daily practice may increase the treatment outcome of esophageal cancer (EC) patients. This retrospective national cohort study assessed the impact on the pathologic complete response (pCR) rate and surgical outcome.

**Patients and Methods:**

Data from EC patients treated between 2009 and 2017 were collected from the national Dutch Upper Gastrointestinal Cancer Audit database. Patients had locally advanced EC (cT1/N+ or cT2-4a/N0-3/M0) and were treated according to the CROSS regimen. CROSS (*n *= 1942) and the extended CROSS (e-CROSS; *n *= 1359) represent patients fulfilling the original or extended CROSS criteria, respectively. The primary outcome was total pCR (ypT0N0), while secondary outcomes were local esophageal pCR (ypT0), surgical radicality, and postoperative morbidity and mortality.

**Results:**

Overall, CROSS and e-CROSS did not differ in total or local pCR rate, although a trend was observed (23.2% vs. 20.4%, *p *= 0.052; and 26.7% vs. 23.8%, *p *= 0.061). When stratifying by histology, the pCR rate was higher in the CROSS group compared with e-CROSS in squamous cell carcinomas (48.2% vs. 33.3%, *p *= 0.000) but not in adenocarcinomas (16.8% vs. 16.9%, *p *= 0.908). Surgical radicality did not differ between groups. Postoperative mortality (3.2% vs. 4.6%, *p *= 0.037) and morbidity (58.3% vs. 61.8%, *p *= 0.048) were higher in e-CROSS.

**Conclusion:**

Extending the CROSS inclusion criteria for neoadjuvant chemoradiotherapy in routine clinical practice of EC patients had no impact on the pCR rate and on radicality, but was associated with increased postoperative mortality and morbidity. Importantly, effects differed between histological subtypes. Hence, in future studies, we should carefully reconsider who will benefit most in the real-world setting.

**Electronic supplementary material:**

The online version of this article (10.1245/s10434-020-09372-y) contains supplementary material, which is available to authorized users.

Neoadjuvant chemotherapy (nCRT) followed by surgery, according to the Chemoradiotherapy for Oesophageal Cancer followed by Surgery Study (CROSS) regimen, increased the 5-year overall survival (OS) in potentially resectable locally advanced esophageal cancer (EC) by 13% compared with surgery alone.^1^ Consequently, nCRT is the current treatment of choice in this patient category in Western Europe.^1^^,^^2^ Inclusion criteria for the CROSS study were potentially curable esophageal or esophagogastric junction carcinoma < 8 cm in length; age ≤ 75 years; adequate hematological, renal, hepatic and pulmonary function; a WHO performance score of ≤ 2; no history of other malignancies; and < 10% weight loss.^2^ However, patients who do not meet the highly selective original CROSS criteria may nonetheless benefit from the CROSS regimen. Over the last decade, the eligibility criteria have been extended in daily practice to all patients with a potentially curative, resectable, locally advanced EC (cT1/N+ or T2-4a/N0-3/M0), if fit for surgery. A recent study (*n *= 161) compared EC patients fulfilling the original CROSS criteria with those who met the extended criteria. Extending these criteria did not affect nCRT toxicity, postoperative complications, or postoperative mortality, but were prognostic for OS.^3^ In another similar study (*n *= 208), no significant differences were found in either survival or in pulmonary, cardiac or anastomotic complications.^4^

In the original CROSS study, 29% of patients treated with nCRT had a pathologic complete response (pCR), which was comparable with rates found in the extended CROSS (e-CROSS) studies.^1^^,^^3^^,^^4^ Achieving pCR after nCRT has a major impact on EC patients, as pCR has been correlated with improved OS.^5^ No standard definition of pCR exists. Some studies have applied the definition of pCR to the locally invasive tumor only (ypT0), while others also included the presence of tumor-free lymph nodes (ypT0N0).^5^^–^8 Moreover, the consistency in defining pCR in studies with patients using the e-CROSS criteria is still questionable and outcomes are contradictory. This multicenter study assessed the influence of extending the CROSS inclusion criteria on both total (ypT0N0) and local (ypT0) pCR in EC patients.

## Patients and Methods

### Patients

For this national multicenter cohort study, we retrieved data from all surgically treated EC patients during the period 2009–2017 in 11 centralized hospitals, as registered retrospectively in the Dutch Upper Gastrointestinal Cancer Audit (DUCA) database. This Surgical Audit Registry only included patients who had surgery with the intent of curative resection, and is mandatory for all these official Dutch EC centers. The DUCA database contains data on patient (e.g. age, sex, and comorbidities), tumor (e.g. histology, cT stage, cN stage, and tumor length), and treatment characteristics [e.g. surgical complications scored according to the Clavien–Dindo classification, type of surgery, postoperative mortality (up to 30 days after surgery or during hospitalization) and re-interventions], as well as pathology (histology, ypT stage, ypN stage, and resection radicality). No long‐term survival data are registered in the DUCA database. This study was conducted with the approval of our local Ethical Board (registration number 2018.588).

In total, 4115 EC patients with an adenocarcinoma or squamous cell carcinoma [cT1/N+ or T2-4a/N0-3/M0 according to the American Joint Committee on Cancer (AJCC) 7th edition TNM staging] 9 who were treated with nCRT followed by surgery, as per the CROSS regimen, were eligible for inclusion. Patients with missing data on pathologic *T* stage (pT) and Mandard tumor regression score (*n *= 4) or on the performed surgical procedure (*n *= 6), and with an irresectable tumor during surgery (*n *= 125) (electronic supplementary Tables 1–4), were excluded. Patients who could not be categorized into either the CROSS or e-CROSS groups due to missing data on the CROSS criteria used were considered as the undefined group (*n *= 679). Finally, 3301 patients were included in one of the CROSS-related groups.

### Methods

Patients were divided into two CROSS-related groups. The CROSS group (*n *= 1942) consisted of patients who met the CROSS inclusion criteria, i.e. tumor length ≤ 8 cm, weight loss < 10%, and age ≤ 75 years, whereas the e-CROSS group (*n *= 1359) consisted of patients who met one or more of the e-CROSS criteria, i.e. tumor longer than 8 cm (*n *= 339), ≥ 10% weight loss (*n *= 784), and/or age ≥ 75 years (*n *= 406). Data regarding other CROSS criteria were not available from the database and could not be evaluated in this study.

The primary outcome was the difference in the total pCR rate, defined as ypT0N0, while secondary outcomes were local or esophageal pCR (ypT0), postoperative morbidity (i.e. complication rate after surgery), postoperative mortality (< 30 days), and microscopic radicality of the surgical resection (R0 vs. R1). In patients with a missing ypT, the Mandard tumor regression grade (TRG) was used. Patients with a Mandard grade of 1 were considered as having pCR, and patients with a Mandard grade of 2–5, i.e. with residual microscopic tumor, were considered as having a non-complete response or as non-responders.

As the original CROSS trial had a predominance of esophageal adenocarcinomas (EACs), but high favorable response in squamous cell carcinoma patients, a separate analysis for each histological subtype was additionally performed.

### Pathology

Histopathologic assessment of the resected esophageal specimen was performed according to the local protocol in the respective medical centers. After hematoxylin and eosin (H&E) staining, the histological tumor type, pathologic T and N stage, and free circumferential resection margins (> 1 mm) were determined by experienced gastrointestinal pathologists.

### Staging

Clinical tumor staging consisted of the assessment of local tumor extension by endoscopy, computed tomography (CT) and endoscopic ultrasound scan (EUS), as well as detection of distant metastases with CT and/or positron emission tomography (PET).

### Treatment

All patients were treated preoperatively according to the CROSS regimen, consisting of five weekly treatments of intravenous carboplatin [area under the curve (AUC) = 2] and paclitaxel (50 mg/m^2^) with concurrent radiotherapy (total dose 41.4 Gy, in daily fractions of 1.8 Gy five times/week), followed by surgical resection within 6–10 weeks after nCRT. Radiotherapy was delivered using a multiple field technique, either the three-dimensional conformal radiation technique or intensity-modulated radiotherapy, depending on the center of treatment. Surgery consisted of a radical open (transthoracic or transhiatal) or minimally invasive esophagectomy with locoregional mediastinal and upper abdominal lymph node dissection.

### Statistical Analysis

Logistic regression and the Chi-square test were used to assess differences between the original CROSS and the e-CROSS groups. Univariate logistic regression analysis was used to assess all possible predictive factors for pCR. All factors with a *p* value < 0.10 in the univariate logistic regression were included in the multivariate logistic regression. A *p* value < 0.05 was considered significant. Statistical analyses were performed using IBM SPSS Statistics for Windows, version 23.0 (IBM Corporation, Armonk, NY, USA).

## Results

### Patient Characteristics

Patient characteristics are described in Table 1. Patients with an irresectable tumor during surgery were excluded (*n* = 125), of whom 45 met the original CROSS criteria, 58 did not, and 22 belonged to the undefined group (*p* = 0.01) (electronic supplementary Tables 1–4). The CROSS (*n *= 1942) and e-CROSS (*n *= 1359) groups differed significantly in tumor localization [mid vs. distal vs. gastroesophageal junction (GEJ); *p *= 0.007], cT stage (cT1–cT4a), and cN stage (cN0–cN3; *p *= 0.000). EAC was the predominant histological type (*n *= 2618) versus esophageal squamous cell carcinoma (ESCC; *n *= 683). Histological subtypes were distributed equally between both groups.

**Table 1:** Patient and tumor characteristics of the CROSS and e-CROSS groups

	CROSS (*n *= 1942)	e-CROSS (*n *= 1359)	*p* Value^a^
Sex, male	1541 (79.4)	1044 (76.8)	0.078
Age, years [median (IQR)]	65 (59–69)	68 (60–76)	*0.000*^b^
Missing	0 (0.0)	4 (0.3)	
*Histology*			0.739
EAC	1544 (79.5)	1074 (79.0)	
ESCC	398 (20.5)	285 (21.0)	
*Tumor location*			*0.007*
Mid	277 (14.3)	183 (13.5)	
Distal	1328 (68.4)	880 (64.8)	
GEJ	333 (17.1)	292 (21.5)	
Missing	4 (0.2)	4 (0.3)	
*Tumor length, cm [median (IQR)]*	4.0 (3.0–6.0)	6.0 (4.0–9.0)	*0.000*^b^
Missing	0 (0.0)	170 (12.5)	
*cT stage*			*0.000*
T1	25 (1.3)	8 (0.6)	
T2	420 (21.6)	205 (15.1)	
T3	1455 (74.9)	1096 (80.6)	
T4a	42 (2.2)	50 (3.6)	
*cN stage*			*0.000*
N0	653 (33.6)	402 (29.6)	
N1	858 (44.2)	562 (41.4)	
N2	337 (17.4)	314 (23.1)	
N3	48 (2.5)	47 (3.5)	
Missing	46 (2.4)	34 (2.5)	

Data are expressed as *n* (%) unless otherwise specified

Italics indicate the name of the main category

*CROSS* Chemoradiotherapy for Oesophageal Cancer followed by Surgery Study, *e-CROSS* extended CROSS, *IQR* interquartile range, *GEJ* gastroesophageal junction, *cT* clinical tumor stage, *cN* clinical node stage, *EAC* esophageal adenocarcinoma, *ESCC* esophageal squamous cell carcinoma

^a^Likelihood ratio test

^b^Mann–Whitney U test

### Pathologic Complete Response (ypT0N0)

In total, 451 (23.2%) patients in the CROSS group had a total pCR (ypT0N0), compared with 277 (20.4%) in the e-CROSS group, which approached significance (*p* = 0.052) (Table 2). Separate analysis of both histological types showed that pCR (ypT0N0) in the ESCC subtype differed significantly between the CROSS and e-CROSS groups (48.2% vs. 33.3%; *p *= 0.000), but not in the EAC subtype (16.8% vs. 16.9%; *p *= 0.908) (Table 3). In univariate logistic regression analyses for pCR, factors with *p* values < 0.1 included the e-CROSS group (*p *= 0.053), sex (*p *= 0.000), clinical T stage (*p *= 0.000), clinical N stage (*p *= 0.042), and histologic type (ESCC vs. EAC; *p *= 0.000). Subsequent multivariate analysis showed clinical T stage (*p* = 0.001) and histology (ESCC; *p *= 0.000) to remain predictive for pCR (Table  4). As our primary interest was in the difference in pCR in the CROSS and e-CROSS groups, we included the variable ‘group’ in the multivariate analysis, which was not predictive for pCR (*p *= 0.090). Assessment of the influence of the individual extensions on total pCR showed that only weight loss (≥ 10%) was predictive in the multivariate analysis (*p *= 0.021) (Table 5). Weight loss ≥ 10% was associated with a lower chance of pCR (odds ratio 0.778, 95% confidence interval 0.629–0.962).

**Table 2:** Complete pathologic response rate in patients in the CROSS and e-CROSS groups

	CROSS (*n *= 1942)	e-CROSS (*n *= 1359)	*p* Value^a^
pCR, ypT0N0	451 (23.2)	277 (20.4)	0.052
pCR, ypT0	519 (26.7)	324 (23.8)	0.061
*ypT stage*			*0.011*
T0	462 (23.8)	294 (21.6)	
T1	342 (17.6)	203 (14.9)	
T2	385 (19.8)	262 (19.3)	
T3	659 (33.9)	544 (40.0)	
T4a	5 (0.3)	7 (0.5)	
T4b	2 (0.1)	2 (0.1)	
Missing	87 (4.5)	47 (3.5)	
*ypN stage*			0.815
N0	1167 (60.1)	810 (59.6)	
N1	410 (21.1)	291 (21.4)	
N2	208 (10.7)	156 (11.5)	
N3	78 (4.5)	62 (4.6)	
Missing	79 (4.1)	40 (2.9)	
*Resection*			0.406
R0	1861 (95.8)	1294 (95.2)	
R1	75 (3.9)	55 (4.0)	
R2	3 (0.2)	3 (0.2)	
Missing	3 (0.2)	7 (0.5)	

Data are expressed as *n* (%)

Italic indicates the name of the main category

*CROSS* Chemoradiotherapy for Oesophageal Cancer followed by Surgery Study, *e-CROSS* extended CROSS, *pCR* pathologic complete response, *ypT0N0* pathologic complete response, *ypT0* pathologic complete local response, *ypT* pathologic tumor stage, *ypN* pathologic node stage, *R0* microscopically radical resection, *R1* microscopically irradical resection margin, *R2* locoregional tumor residue

^a^Likelihood ratio test

**Table 3:** Separate analysis of esophageal adenocarcinoma and esophageal cell carcinoma

	Esophageal adenocarcinoma		*p* Value^a^
	CROSS (*n *= 1544)	e-CROSS (*n *= 1074)	
pCR, ypT0N0	259 (16.8)	182 (16.9)	0.908
pCR, ypT0	298 (19.3)	207 (19.3)	0.986
*Resection*			0.457
R0	1477 (95.7)	1015 (94.5)	
R1	62 (4.0)	51 (4.7)	
R2	2 (0.1)	2 (0.2)	
Missing	3 (0.2)	4 (0.4)	
Postoperative morbidity	881 (57.1)	656 (61.1)	*0.042*
Postoperative mortality (< 30 days)	44 (2.8)	44 (4.1)	0.090

	*Esophageal squamous cell carcinoma*		*p* Value^a^
	CROSS (*n *= 398)	e-CROSS (*n *= 285)	
pCR, ypT0N0	192 (48.2)	95 (33.3)	*0.000*
pCR, ypT0	221 (55.5)	117 (41.1)	*0.000*
*Resection*			0.229
R0	384 (96.5)	279 (97.9)	
R1	13 (3.3)	4 (1.4)	
R2	1 (0.3)	1 (0.4)	
Missing	0 (0.0)	1 (0.4)	
Postoperative morbidity	252 (63.3)	184 (64.6)	0.738
Postoperative mortality (< 30 days)	18 (4.5)	19 (6.7)	0.227

Data are expressed as *n* (%)

Italics indicate the name of the main category

*CROSS* Chemoradiotherapy for Oesophageal Cancer followed by Surgery Study, *e-CROSS* extended CROSS, *pCR* pathologic complete response, *ypT0N0* pathologic complete response, *ypT0* pathologic complete local response

^a^Likelihood ratio test

**Table 4:** Univariate and multivariate logistic regression of patient and tumor characteristics

	pCR method 1 (ypT0N0)		pCR method 2 (ypT0)	
	OR (95% CI)	*p* Value	OR (95% CI)	*p* Value
*Univariate logistic regression analysis*				
Extended CROSS	0.846 (0.715–1.002)	0.053	0.858 (0.731–1.007)	0.062
Female	1.675 (1.388–2.020)	*0.000*	1.821 (1.523–2.178)	*0.000*
ESCC	3.578 (2.979–4.297)	*0.000*	3.099 (3.429–4.901)	*0.000*
cT1 and cT2	1.000	*0.000*^a^	1.000	*0.001*^a^
cT3	0.672 (0.553–0.817)	*0.000*	0.711 (0.589–0.858)	*0.000*
cT4	0.542 (0.308–0.955)	*0.034*	0.501 (0.288–0.870)	*0.014*
cN0	1.000	*0.042*^a^	1.000	0.409^a^
cN1	0.807 (0.668–0.974)	*0.026*	0.874 (0.729–1.048)	0.147
cN2	0.773 (0.611–0.979)	*0.033*	0.890 (0.712–1.112)	0.304
cN3	0.610 (0.350–1.063)	0.081	0.756 (0.457–1.250)	0.275
*Multivariate logistic regression analysis*				
Extended CROSS	0.926 (0.847–1.012)	0.090	0.861 (0.728–1.019)	0.081
Female	1.089 (0.883–1.343)	0.424	1.249 (1.045–1.494)	*0.015*
ESCC	3.575 (2.932–4.360)	*0.000*	3.774 (3.176–4.484)	*0.000*
cT1 and T2	1.000	*0.001*^a^	1.000	*0.000*^a^
cT3	0.696 (0.565–0.859)	*0.001*	0.716 (0.601–0.853)	*0.000*
cT4	0.494 (0.272–0.899)	*0.021*	0.419 (0.245–0.717)	*0.001*
cN0	1.000	0.222^a^		
cN1	0.832 (0.682–1.014)	0.069		
cN2	0.854 (0.664–1.098)	0.219		
cN3	0.678 (0.381–1.205)	0.185		

Italics indicate the name of the main category

*pCR* pathologic complete response, *CI* confidence interval, *OR* odds ratio, *ESCC* esophageal squamous cell carcinoma, *cN* clinical lymph node stage, *cT* clinical tumor stage, *CROSS* Chemoradiotherapy for Oesophageal Cancer followed by Surgery Study

Variables with a *p* value <0.10 on univariate analysis were included in the multivariate analysis

^a^Overall *p* Value

**Table 5:** Univariate and multivariate logistic regression of individual CROSS criteria

	pCR method 1 (ypT0N0)		pCR method 2 (ypT0)	
	OR (95% CI)	*p* Value	OR (95% CI)	*p* Value
*Univariate logistic regression analysis*				
Tumor length > 8 cm	0.835 (0.630–1.107)	0.209	0.839 (0.643–1.096)	0.198
Weight loss ≥ 10%	0.821 (0.672–1.003)	0.054	0.874 (0.724–1.054)	0.159
Age ≥ 75 years	0.895 (0.692–1.157)	0.396	0.875 (0.685–1.117)	0.285
*Multivariate logistic regression analysis*				
Tumor length >8 cm	0.957 (0.710–1.290)	0.773	0.907 (0.686–1.201)	0.496
Weight loss ≥ 10%	0.778 (0.629–0.962)	*0.021*	0.833 (0.683–1.016)	0.071
Age ≥ 75 years	0.941 (0.720–1.229)	0.941	0.892 (0.693–1.153)	0.386

Italic indicates the name of the main category

*CROSS* Chemoradiotherapy for Oesophageal Cancer followed by Surgery Study, *pCR* complete pathologic response, *CI* confidence interval, *OR* odds ratio

### Pathologic Complete Local Response (ypT0)

The proportion of patients with a local pCR (ypT0) did not differ (*p *= 0.061) between the CROSS (*n *= 519, 26.7%) and e-CROSS (*n *= 324, 23.8%) groups (Table 2). Separate analysis of both histological types showed a significant difference in local pCR (ypT0) between the CROSS and e-CROSS groups (55.5% vs. 41.1%; *p *= 0.000) in the ESCC subtype, but not in the EAC subtype (19.3% vs. 19.3%, respectively; *p *= 0.986). Upon univariate logistic regression, clinical T stage, sex, and histology had *p* values < 0.1. In the subsequent multivariate logistic regression, clinical T stage (*p *= 0.000), sex (female; *p *= 0.015), and histology (ESCC; *p *= 0.000) were predictive for local pCR (Table 4). The variable ‘group’ was included in the multivariate analysis as this was the explanatory variable of primary interest. Extending the CROSS criteria showed not to be predictive for pCR (*p *= 0.081). Moreover, none of the individual extended criteria were predictive for local pCR in univariate and multivariate analyses (Table 5).

### Surgical Radicality, Postoperative Mortality and Morbidity

The proportion of patients with a curative radical (R0) resection was not different between both groups (CROSS 95.8% vs. e-CROSS 95.2%; *p *= 0.406) (Table 1). Postoperative mortality (< 30 days) was significantly higher in the e-CROSS group than in the CROSS group [63 (4.6%) vs. 62 (3.2%); *p *= 0.037] (Table 6), and postoperative morbidity was also higher in the e-CROSS group than in the CROSS group, i.e. 840 (61.8%) vs. 1133 (58.3%) (*p *= 0.048). Specifically, readmission to the intensive care unit (ICU) was higher in the e-CROSS group than in the CROSS group, i.e. 206 (15.2%) vs. 235 (12.1%) (*p *= 0.011). The two groups did not differ in pulmonary complications (*p *= 0.534), ICU stay (*p *= 0.091), re-intervention (*p *= 0.405), need for re-intervention under general anesthesia (*p *= 0.111), and hospital readmission (*p *= 0.517). Separate analyses in the EAC and ESCC subgroups showed no differences in postoperative mortality (Table 3), whereas postoperative morbidity was significant higher in the EAC subtype of the e-CROSS group (CROSS 57.1% vs. e-CROSS 61.1%; *p *= 0.042), but not in the ESCC subtype (63.3% vs. 64.6%; *p *= 0.738).

**Table 6.** Postoperative complications

	CROSS (*n *= 1942)	e-CROSS (*n = *1359)	*p* Value^a^
Postoperative mortality < 30 days	62 (3.2)	63 (4.6)	*0.037*
Missing	14 (0.7)	2 (0.1)	
*Postoperative morbidity*			*0.048*
Yes	1133 (58.3)	840 (61.8	
No	806 (41.5)	518 (38.1%)	
Missing	3 (0.2)	1 (0.1)	
Pulmonary complications	592 (30.5)	426 (31.3)	0.534
Missing	809 (41.7)	521 (38.3)	
Need for reintervention	452 (23.3)	334 (24.6)	0.405
Missing	8 (0.4)	3 (0.2)	
Need for reintervention under general anesthesia	62 (3.2)	43 (3.2)	0.111
Missing	1825 (94.0)	1276 (93.9)	
Days in the ICU [median (IQR)]	2 (1–4)	2 (1–4)	0.091
Missing	182 (9.4)	143 (10.5)	
Readmission to the ICU	235 (12.1)	206 (15.2)	*0.011*
Missing	5 (0.3)	5 (0.4)	
Readmission to hospital	256 (13.2)	190 (14.0)	0.517
Missing	22 (1.1)	14 (1.0)	

Data are expressed as *n* (%) unless otherwise specified

Italics indicate the name of the main category

*CROSS* Chemoradiotherapy for Oesophageal Cancer followed by Surgery Study, *e-CROSS* extended CROSS, *IQR* interquartile range, *ICU* intensive care unit

^a^Likelihood ratio test

## Discussion

Since the CROSS trial, criteria for nCRT have been expanded to treat more patients with clinically resectable, locally advanced EC (cT1/N+ or cT2-4a/N0-3/M0); however, the efficacy and tolerability of the CROSS regimen in patients not fulfilling the original study criteria seems to be controversial.^10^^–^12 In this retrospective study, we evaluated the effect of extending the CROSS inclusion criteria on pCR in a nationwide, real-world, multicenter cohort. Extension of the original CROSS inclusion criteria for nCRT in EC patients had no impact on the pCR rate overall. Interestingly, separate analyses of the EAC and ESCC histological subtypes showed a significantly lower pCR rate in ESCC patients in the e-CROSS group compared with the CROSS group. On the other hand, postoperative mortality and morbidity were higher in EC patients who did not meet the initial CROSS criteria (Table 6).

In this large-scale, nationwide study, we found a total pCR (ypT0N0) rate of 23.2% in the group fulfilling the original CROSS criteria, and 20.4% in those fulfilling the e-CROSS criteria. The pCR rate in the CROSS group in our study was lower than the 29% in the original CROSS study and a study in Rotterdam that found a pCR (ypT0N0) rate of 27% in the CROSS group and a rate of 28% in the e-CROSS group.^4^ The pCR rates might differ due to different definitions of pCR using either ypT0N0 (total) or ypT0 (local) as the standard, as well as differences in tumor histology. Two other previous studies report pCR rates of 41% and 15% in patients fulfilling the CROSS criteria;^13^^,^^14^ however, both studies used different start populations, consisting of 75% ESCC, compared with the 20.5% of ESCC patients in our CROSS group, or included only adenocarcinoma patients, of whom 13% had distant metastases. Indeed, the pCR rates in the CROSS group in the separated ESCC and EAC analysis (48.2% and 16.8%, respectively) were shown to be similar to the studies of Soror et al.^13^ and Lorenzen et al.^14^, respectively, and are in line with the pCR rates for ESCC being more favorable compared with the pCR rates for EAC. Moreover, diagnostic options nowadays are more advanced using EUS and PET/CT, whereas only EUS and CT scans were used in the original CROSS criteria.^1^ This might affect pCR indirectly due to differences in the inclusion of patients. Differences between our study and that of other studies might also be explained by the different histopathological protocols of sectioning the surgical specimens, diagnosing complete response using only H&E staining, and/or using immunohistochemistry and interobserver variation. Different individual criteria such as weight loss, age, and tumor length might also explain the different pCR rates. The study by Lorenzen et al. showed that weight loss was an independent factor for achieving pCR;^14^ however, in that study, any weight loss was considered as ‘positive’ instead of a range (i.e. 10% cut-off for weight loss) in the CROSS criteria, which underlies our finding of a worse total pCR in the e-CROSS patients. Furthermore, tumor length and older age were regarded as predictive factors that increase the pCR rate;^11^ however, many studies adhere to different tumor lengths (according to the 5th edition of the AJCC staging manual) and age standards, which do not match the CROSS criteria. Therefore, it was not possible to compare these findings using the current study.

Other factors that were predictive for pCR (ypT0N0) in multivariate logistic regression included cT stage and ESCC as the tumor type. These outcomes were in line with several studies in which ESCC was more likely to achieve pCR.^5^^,^^15^ Likewise, ESCC and lower clinical T stage, as well as female sex, were associated with local pCR (ypT0). Due to a lack of literature assessing the effect of nCRT according to the CROSS regimen on local pCR, these findings were not able to be compared with other studies.

We showed that patients in the e-CROSS group had a significantly higher postoperative complication rate than patients in the CROSS group, specifically in the EAC subgroup, although a similar trend was observed in the smaller ESCC subgroup. This might indicate that other therapeutic options such as definitive chemoradiotherapy (dCRT) could be considered, as it has been shown to be an alternative for patients who are not fit enough to participate in CROSS.^16^ On the contrary, the study of De Heer et al. found no differences in postoperative complications between patients in the CROSS and e-CROSS groups.^3^ Although the difference in postoperative complications between CROSS and e-CROSS is small in both studies, the fact that our study showed a significant result in the postoperative complication rate is likely due to the larger sample size.

Limitations of this study include the fact that the DUCA database did not contain data regarding other CROSS eligibility criteria, i.e. the presence of celiac lymph node metastases, tumor extension in the gastric cardia, and WHO performance status < 2. The original CROSS study excluded patients who had celiac lymph node metastases, as these metastases were previously classified as M1a according to AJCC 6th edition TNM staging manual;^1^ however, according to the AJCC 7th edition TNM staging manual, celiac lymph node metastases are classified as N+. In the literature, the prognostic value of celiac lymph node metastases is unclear,^15^ therefore it is important to assess the effects of extending the CROSS criteria specifically in patients with celiac lymph node metastases. The exclusion of patients with missing CROSS eligibility data has led to a loss of 17% of patients (*n *= 679; the undefined group). Due to the uncertainty of the CROSS criteria, this undefined group was excluded in order to prevent skewed results; however, the group was used in an additional analysis, along with the CROSS and e-CROSS groups, to check the impact of excluding 17% of patients. Excluding the undefined group did not result in a significant difference in the results of this study and was therefore not taken into consideration. Another limitation was that patients with an irresectable tumor (*n *= 125) were excluded from this study, which may have contributed to a higher proportion of pCRs. Inclusion of these patients with irresectable tumors would have led to a 22.7% pCR (ypT0N0) rate for the CROSS group and 19.5% for the e-CROSS group (*p *= 0.035) (electronic supplementary Table 2). Future validation of our findings should focus on prospective studies including more CROSS criteria and irresectable tumors.

## Conclusion

This nationwide, multicenter, retrospective study shows that extending the CROSS criteria is not associated with a significant difference in pCR rates, although a trend towards a lower pCR rate was observed in patients meeting the extended criteria. However, separate analysis in the histological subtypes showed a significantly lower pCR rate for the e-CROSS group in ESCC. In addition, postoperative mortality and morbidity were higher in patients not fulfilling the original CROSS criteria. Weight loss > 10% was the only individual criterion to have an impact on pCR. Therefore, future prospective studies should focus on the effect of individual extensions of the CROSS criteria on OS, and explore alternative treatment options for preventing complications, such as dCRT.

## Electronic supplementary material

Below is the link to the electronic supplementary material.

Supplementary file1 (DOCX 31 kb)
