# Supplementary material for: Effect of Extending the Original CROSS Criteria on Tumor Response to Neoadjuvant Chemoradiotherapy in Esophageal Cancer Patients: A National Multicenter Cohort Analysis
Source: Ann Surg Oncol. 2020 Nov 28;28(7):3951–60. doi: 10.1245/s10434-020-09372-y (PMC8184698; doi:10.1245/s10434-020-09372-y)
Supplement: Supplementary file 1 — Supplementary file1 (DOCX 31 kb) [file 10434_2020_9372_MOESM1_ESM.docx]

**Supplementary table 1**. **Patient and tumor characteristics of group-I (CROSS inclusion criteria), group-II (extended CROSS criteria) including irresectable patients (*n=*125*)***

|  | Group I (*n*=1987), *n*(%) | Group II (*n*=1417), *n*(%) | *P*-value^a^ |
| --- | --- | --- | --- |
| Gender (Male) | 1579 (79.5%) | 1081 (76.3%) | 0.082 |
| Age (years) (median) | 65 (59-69) | 68 (60-76) | *0.000^b^* |
| Histology  Adenocarcinoma  Squamous cell carcinoma | 1575(79.3%)  412 (20.7%) | 1122 (79.2%)  295 (20.8%) | 0.712 |
| Tumour location  Mid  Distal  GEJ  Missing | 288 (14.5%) 1349 (67.9%) 346 (17.4%)  4 (0.2%) | 189 (13.3%) 912(64.4%) 312 (22.0%)  4 (0.3%) | *0.003* |
| Tumor length (cm) (median) | 4.0 (3.0-6.0) | 6.0 (4.0-9.0) | *0.000 ^b^* |
| cT stage  T1  T2  T3  T4a | 25 (1.3%)  427 (21.5%)  1490 (75.0%)  45 (2.3%) | 8 (0.6%)  210 (14.8%)  1143 (80.7%)  56 (4.0%) | *0.000* |
| cN-stage  N0  N1  N2  N3  Missing | 661 (33.3%)  883 (44.4%)  345 (17.4%)  50 (2.5%)  48 (2.4%) | 415 (29.3%)  591 (41.7%)  328 (23.1%)  47 (3.3%)  36 (2.5%) | *0.000* |

Abbreviations: IQR = interquartile range, GEJ = gastro-esophageal junction, cT = clinical tumor stage, cN = clinical node stage

^a^. Likelihood ratio test
^b^. Mann-Whitney-U test

**Supplementary table 2. Pathologic complete response rate in patients of group-I and II, including irresectable patients**

|  | Group I (*n*=1987), *n*(%) | Group II (*n*=1417), *n*(%) | *P*-value^a^ |
| --- | --- | --- | --- |
| pCR (ypT0N0) | 451 (22.7%) | 277 (19.5%) | *0.035* |
| pCR (ypT0) | 519 (26.1%) | 324 (22.9%) | *0.045* |
| ypT-stage  T0  T1  T2  T3 T4a  T4b  Missing | 462 (23.3%)  342 (0.17%)  385 (0.19%)  660 (33.2%)  5 (0.3%)  2 (0.1%)  131 (6.6%) | 294 (20.7%)  203 (14.3%)  262 (18.5%)  544 (38.4%)  7 (0.5%)  2 (0.1%)  105 (7.4%) | *0.013* |
| ypN stage  N0  N1  N2  N3  Missing | 1167 (58.7%)  410 (20.6%)  208 (10.5%)  78 (3.9%)  124 (6.2%) | 810 (57.2%)  291 (20.5%)  156 (11.0%)  62 (4.4%)  98 (6.9%) | 0.337 |
| Resection  R0  R1  R2  Unknown  Missing | 1861 (93.7%)  75 (3.8%)  3 (0.2%)  3 (0.2%)  45 (2.3%) | 1294 (91.3%)  55 (3.9%)  3 (0.2%)  6 (0.4%)  59 (4.2%) | 0.477 |

Abbreviations: pCR = pathologic complete response, ypT0N0 = pathologic complete response, ypT0 = pathologic complete local response, ypT = pathologic tumor stage, ypN = pathologic node stage, R0 = microscopically radical resection, R1 = microscopically irradical resection margin, R2 = locoregional tumor residue

^a^. Likelihood ratio test

**Supplementary table 3. Univariate and multivariate logistic regression of patient and tumor characteristics, including irresectable patients**

| **Univariate logistic regression analysis** | | | | | | | | | |
| --- | --- | --- | --- | --- | --- | --- | --- | --- | --- |
|  | | pCR (ypT0N0) | | | | | pCR (ypT0) | | |
|  | | OR (95% CI) | | | *P*-value | | OR (95% CI) | | *P*-value |
| Extended CROSS | | 0.828 (0.699-0.979) | | | *0.027* | | 0.838 (0.715-0.983) | | *0.030* |
| Female | | 1.66 (1.410-1.970) | | | *0.000* | | 1.789 (1.52-2.10) | | *0.000* |
| SCC | | 3.304 (2.801-3.898) | | | *0.000* | | 3.808 (3.243-4.472) | | *0.000* |
| cT1 & T2 cT3 cT4 | | 1.000  0.654 (0.550-0.777)  0.531 (0.317-0.890) | | | *0.000**  *0.000*  *0.016* | | 1.000  0.699 (0.592-0.826)  0.491 (0.296-0.814) | | *0.000**  *0.000*  *0.006* |
| cN0  cN1  cN2  cN3 | | 1.000  0.821 (0.694-0.972)  0.741 (0.596-0.920)  0.597 (0.359-0.991) | | | *0.011**  *0.022*  *0.007*  *0.046* | | 1.000  0.868 (0.738-1.021)  0.876 (0.714-1.073)  0.701 (0.440-1.116) | | 0.206*  0.088  0.200  0.134 |
| **Multivariate logistic regression analysis** | | | | | | | | | |
|  | pCR method 1 (ypT0N0) | | | pCR method 2 (ypT0) | | | | | |
|  | OR (95% CI) | | *P*-value |  | | OR (95% CI) | | *P*-value | |
| Extended CROSS | 0.850 (0.713-1.014) | | 0.071 | Extended CROSS | | 0.849 (0.718-1.002) | | 0.053 | |
| Female | 1.154 (0.958-1.390) | | 0.133 | Female | | 1.231 (1.032-1.469) | | *0.021* | |
| SCC | 3.281 (2.746-3.920) | | *0.000* | SCC | | 3.683 (3.106-4.368) | | *0.000* | |
| cT1 & T2  cT3  cT4 | 1.000  0.684 (0.568-0.823) 0.488 (0.283-0.841) | | *0.000* 0.000*  *0.010* | cT1 & T2  cT3  cT4 | | 1.000  0.697 (0.586-0.830) 0.373 (0.220-0.634) | | *0.000* 0.000*  *0.000* | |
| cN0  cN1  cN2  cN3 | 1.000 0.841 (0.705-1.004)  0.815 (0.648-1.025)  0.634 (0.375-1.071) | | 0.098*  0.055  0.080  0.089 |  | |  | |  | |

*overall *P*-value

Variables with *P* < 0.10 on univariate analysis were included in the multivariate analysis. Abbreviations: pCR = pathologic complete response, CI = confidence interval, OR = odds ratio, SCC = squamous cell carcinoma, cN = clinical lymph node stage, cT = clinical tumor stage

**Supplementary table 4. Post-operative complications, including irresectable patients**

|  | Group I (*n*=1987), *n*(%) | Group II (*n*=1417), *n*(%) | *P*-value^a^ |
| --- | --- | --- | --- |
| Post-operative mortality <30 days | 63 (3.2%) | 71 (5.0%) | *0.003* |
| Postoperative morbidity  Yes  No  Pulmonary complications  Missing | 1140 (57.4%)  843 (42.5%)  597 (52.4%)  847 (42.6%) | 858 (60.6%)  557 (39.4%)  438 (31.0%)  561 (39.6%) | 0.134  0.347 |
| Need for reintervention | 454 (22.8%) | 339 (23.9%) | 0.666 |
| Need for reintervention under general anesthesia | 63 (3.2%) | 43 (3.0%) | 0.595 |
| Readmission ICU | 237 (11.9%) | 208 (14.7%) | 0.034 |
| Readmission hospital | 259 (0.13%) | 191 (13.5%) | 0.275 |

IQR = interquartile range, ICU = intensive care unit

^a^. Likelihood ratio
